# Perceptions of Organizational Justice Among Nurses Working in University Hospitals of Shiraz: A Comparison Between General and Specialty Settings

**DOI:** 10.5812/nms.10637

**Published:** 2013-12-09

**Authors:** Nahid Hatam, Mozhgan Fardid, Zahra Kavosi

**Affiliations:** 1 Department of Health Management, School of Management and Medical Information Sciences, Shiraz University of Medical Sciences, Shiraz, IR Iran; 2 Student Research Committee, School of Management and Medical Information Sciences, Shiraz University of Medical Sciences, Shiraz, IR Iran

**Keywords:** Social Justice, Health Manpower, Nurses, Hospitals, General Practice

## Abstract

**Background:**

Justice has gained much attention in social and human studies and has many consequences on employees and the organizations, especially on health system workers such as nurses who are among the key factors in health care systems.

**Objectives:**

The purpose of this study was to investigate perception of organizational justice among nurses in educational hospitals of Shiraz University of Medical Sciences (SUMS), and to compare the results of general and specialty hospitals.

**Materials and Methods:**

In this research, 400 nurses at SUMS hospitals were selected by random sampling method. A 19-item questionnaire was applied to measure distributive, procedural and interactional justice. Data analysis was performed using descriptive statistics, including percentage, frequency, mean, and standard deviation. Also, the t-test and one way ANOVA were used to measure the differences between different hospitals and wards.

**Results:**

Of 400 nurses, 66% perceived a high level of organizational justice. In this study the mean scores of total perceived organizational justice (P = 0.035), procedural justice (P = 0.031), and interactional justice (P = 0.046) in specialty hospitals were higher than general ones. Furthermore, the mean score of interactional justice was higher than the other components of organizational justice, respectively 3.58 ± 1.02 for general and 3.76 ± 0.86 for specialty hospitals. Significant differences were observed between overall perceived justice (P = 0.013) and its components (P = 0.024, P = 0.013, and P = 0.036) in different wards.

**Conclusions:**

Most nurses who participated in this study had a high perception of organizational justice. The mean score of organizational justice was higher in specialty hospitals. Health care policy makers and hospital managers should support their employees, especially nurses through fairness in distributions, procedures, and interactions.

## 1. Background

The most important part of each organization is its human resource. The way managers behave and treat staff would affect their attitudes and working behaviors (1). When people have a positive attitude toward their job, their manager, department or organization they work in, they become much more motivated to work efficiently (2). Nowadays, organizations are faced with educated staff, who are not only searching for better jobs, but also expecting more respect (1). This issue is more important for healthcare organizations than other ones. Nurses as an indispensable component of the work force in the healthcare system (3), constitute the largest professional group in the hospital, and spend most of their lives over there (4). In fact, nurses as the frontline workers of the hospitals have a great effect on the patients’ point of views and the quality of care (5). On the other hand, organizational justice (OJ) is a key variable to promote effectiveness in organizations as a competitive advantage. Previous studies suggest that people attitude is affected by their perceptions of OJ (6).The results of a study on 285 employees in the US showed that OJ affects individual’s attitudes such as job satisfaction (7). OJ has been a popular field of study in the social sciences for decades (8), and is one of the most popular research areas in organizational behavior (6). Generally organizational justice is defined as the conditions in which employees believe that their organization is treating them fairly or unfairly (9). The findings of previous studies indicated the significant effect of OJ on employees’ behaviors, attitudes, job satisfaction (6, 9-11), commitment (12, 13), trust (2, 10), organizational citizenship behaviors (OCBs) (11, 14, 15), well-being and performance (16), and organizational outcomes and negative reactions such as staff turnover (8, 13, 17, 18). By perceiving unfairness, personnel’s morality declines up to leaving their jobs or even working against the organization (9). Conlon et al. in their review on the effect of OJ on staff performance, categorized these behaviors as “the good” like task performance, “the bad” such as turnover, and “ugly” such as behaviors against the organization (19). Heponiemi et al. reported that OJ perception is important because it acts as a buffer against undesirable effects of a number of detrimental factors like interference with family life (6, 20). Thus, in highly demanding and stressful situations of healthcare services, feeling a high level of OJ may help employees to cope with such a situation (6). Based on individuals’ perceptions of fairness within their organization, three kinds of OJ have been defined including the distributive, procedural, and interactional justice (21). Distributive justice is the perceived fairness of outcome distributions. People usually compare their output with their input and compare this ratio with that of their colleagues in their organization or also in other organizations which are approximately in the same condition (9). A distribution would be perceived fair only if it is consistent with the rules of allocation (22, 23). When there is unfairness in the organization, inefficient workers would do their job even worse (24). Procedural Justice refers to the fairness of the process which leads to the outcomes. This issue is known as the “voice phenomenon” which means that people feel fairness when they can make a “voice” in the process instead of being “mute” (25). Studies have shown that when employees feel fairness in the process of resource allocation, they reciprocate this social reward in the form of OCBs (14, 15). The third dimension of justice, interactional justice means that people consider the fairness in how they are treated by others as well (15). Considering the vital role of nurses in people’s health improvement, lack of justice perception and its unwanted and detrimental consequences is an important issue to be studied. Studies have found that perception of discrimination is associated with job dissatisfaction, lack of organizational commitment, and intention to leave (13, 25, 26). Recently the hospital industry has been expanded by rapid growth of specialty hospitals. Shiraz University has 15 hospitals of which, 13 are special and only two are general. Specialty hospitals provide care to a special group of patients and their grace is more than general counterparts in attracting nurses. Proponents of such hospitals cite specialization as an opportunity for care improvement; while the opponents believe that these settings seek low-risk patients (27). Despite these concerns, there are limited data comparing specialty and general hospitals (28), and to our knowledge no study has been performed to compare OJ between general and specialty hospitals. Also, only a few studies have investigated the role of all three kinds of OJ (13); while it seems that they all have effects on employees’ attitudes and behaviors. 

## 2. Objectives

This study was performed to determine and compare perceived OJ of nurses in general and specialty hospitals of SUMS. The following hypotheses were analyzed:

- Perceptions of organizational justice in general and specialty hospitals are different regarding gender, and job rank.
- Nurses’ perceptions of total organizational justice and its subscales in general and specialty hospitals are different.
- Nurses working in different wards have different perceptions of organizational justice.

## 3. Materials and Methods

A cross-sectional study was conducted on nurses working at 15 educational hospitals affiliated to SUMS. Cochran formula (29, 30) was used to determine the minimum sample size. Then 325 samples were estimated to be needed based on the following parameters from a total of 2095 nurses working in the mentioned university hospitals (α = 0.05, N = 2095, p = 0.5, q = 0.5, Sampling error = 0.05, Ratio of the departure of an estimated parameter from its notional value and its standard error =1.96). However, 580 samples were entered the study for increasing the validity, and to compensate a possible attrition rate.

The needed number of nurses from each hospital was calculated by dividing the total number of nurses by the estimated sample size. Then, stratified random sampling was performed in each hospital, considering each ward as a stratum. The subjects in each stratum were randomly selected using the list of nurses in each ward. The nurses were informed by the nursing services administration of the hospital about the objectives of the study, and that their participation was voluntary. The inclusion criteria were as follows: working as a nurse or a nurse aid, having a nursing diploma or higher nursing qualifications, having at least one year of experience in nursing, and willingness to participate. The age did not matter in this study. The instrument used in this study was consisted of two sections. The first section included questions about demographics including age, gender, education level, marital status, job rank, ward name, years of experience in nursing, number of children, and the age of the youngest child. The second part of the instrument was the “Organizational Justice Questionnaire’’ developed by Niehoff and Moorman translated to Persian by Moghimi and Ramezan (31). The questionnaire had 19 items; the first five-item measured the distributive Justice; the second five-item measured the procedural Justice, and the other nine items measured interactional justice. The response format was a five-point Likert-type scale ranging from one (I strongly disagree) to five (I strongly agree). Content validity of the Persian questionnaire was confirmed by gathering and using the comments of six faculty members in SUMS. Besides, the overall Cronbach’s alpha for the instrument was calculated as 0.95 for the total OJ questionnaire, and 0.94, 0.86, and 0.94 for the distributive, procedural and interactional justice, respectively. Furthermore, scale reliability was measured by confirmatory factor analysis as 0.70, 0.80, and 0.84 for the mentioned subscales. The nurses answered the questions at work and lonely.

### 3.1. Data Analysis

Data was analyzed using Statistical Package for the Social Sciences (SPSS) software version 16.0 at 0.05 alpha levels. Descriptive statistics, including percentage, frequency, mean, and standard deviation were used to report the demographic characteristics, and mean scores of OJ scale and its subscales. The questions of each subscale were added together and divided by the number of questions in that subscale, so the mean score of each subscale was reported by a number between one and five. The cut-off point of three of five was used in data analysis. Then, the average equal or more than three, was considered as having a perception of high level OJ, and the mean less than three was considered as perception of low level of OJ. The results were also provided through analytical statistics using t-test for analyzing the differences between general and specialty hospitals. Since there were more than two wards in each hospital; one way ANOVA was used to assess the differences among wards regarding OJ perception of nurses.

### 3.2. Ethical Considerations

This paper was extracted from a master thesis, and its proposal had been approved by the vice chancellor for research affairs, ethics committee, and the vice chancellor for education of the management school at SUMS. Then the formal license was obtained from all university hospitals. The nurses were informed that their participation was voluntary and confidential. All the nursing staff were invited to participate without any obligations. The study questionnaire was included in a packet and distributed to participants between June and September 2012. The packet contained the questionnaire, a letter describing the purposes of the study, and assurance that participation in the study was voluntary, and also a written informed consent to be signed.

## 4. Results

From a total of 580 questionnaires, 404 were returned. Four incomplete questionnaires were discarded, and 400 fully completed questionnaires were entered the study. Most of the respondents (n = 351, 87.8%) were female. The predominant age range was 26 to 30 years old (n = 157, 38.9%). Most respondents (n = 341, 85.3%) had a bachelor degree or higher. Nearly a half (n = 237, 59.3%) were married, and the tenure of a half (n = 202, 50.5%) was less than five years. Most participants were nurses (n = 326, 81.5%), and the others were nurse aids (n = 57, 14.3%), and head nurses (n = 17, 4.3%), respectively. The comparison between nurses of general and specialty hospitals showed that in both settings, female, high educated, and married ones were dominant. In the current study, most of nurses (66.3%) had a perception score equal or higher than the cut-off point in fairness perceptions; while, the rest (33.7%) obtained a score below the cut-off point. Table 1 shows that the mean score of perceived OJ was higher in male nurses. A statistically significant difference was observed between the females' mean score of perceived justice in the two settings (P = 0.041). 

**Table 1.** Differences of Organizational Justice Between General and Specialty Hospitals Regarding Gender, Education, and Job Rank

Variable	Kind of Hospital, No. (%)		Score of Organizational Justice in Different Hospitals, Mean (SD)		P value
	General	Specialty	General	Specialty		
**Gender**					
Male	21 (11)	28 (14)	3.44 (0.85)	3.50 (0.67)	0.77
Female	174 (89)	177 (86)	3.14 (0.89)	3.33 (0.76)	0.04
**Education**					
Diploma	34 (17.4)	25 (12.2)	3.22 (1.01)	3.60 (0.86)	0.09
Bachelor and above	161 (82.6)	180 (87.8)	3.16 (0.68)	3.31 (0.76)	0.09
**Job Rank**					
Nurse's aide	32 (16.4)	25 (12.2)	3.35 (0.90)	3.60 (0.68)	0.26
Nurses and head nurses	163 (83.6)	180 (87.8)	3.14 (0.88)	3.31 (0.76)	0.05

Results also showed that the mean of OJ among nurse aids was higher than those working as a nurse or head nurse, but this difference was not significant regarding the work settings (i.e. general and specialty settings). In addition, the mean of total OJ, procedural justice and interactional justice were significantly higher in nurses working in specialty settings than those working in general ones (Table 2). Also, as Table 3 shows, the perception of justice was different among different wards. To identify any differences between nurses' perception of justice in different wards, post-hoc Tukey test was used, and the results (Table 4) showed that perception of justice were different between nurses in surgical wards and other units (i.e. Emergency, critical care, Oncology, Burn, Transplant) (P < 0.05). 

**Table 2.** Comparison between the Mean Score of Organizational Justice and Its Different Aspects in the Studied Subjects of General and Specialty Settings

Scales	Hospitals		95% CI of the Difference		Sig	t
	General, n = 195, Mean (SD)	Specialty, n = 205, Mean (SD)	Lower	Upper		
**Overall organizational justice**	3.17 (0.89)	3.35 (0.75)	-0.33	-0.01	0.04	-2.12
**Distributive justice**	2.43 (1.03)	2.55 (0.94)	-0.31	0.07	0.23	-1.20
**Procedural justice**	3.20 (0.98)	3.40 (0.91)	-0.39	0.02	0.03	-2.16
**Interactional justice**	3.58 (1.02)	3.76 (0.86)	-0.37	-0.003	0.05	-2.003

**Table 3.** Differences in Perceptions of Justice Among Nurses Working in Different Wards of Hospitals

	Surgical, Mean (SD)	Internal Medicine, Mean (SD)	Critical Care^a^, Mean (SD)	Neurology, Oncology, Burn, Transplant, Mean (SD)	Statistical Test, F	Statistical Test, P value
**Overall organizational justice**	3.46 (0.87)	3.26 (0.70)	3.17 (0.81)	3.06 (0.75)	4.80	0.003
**Distributive justice**	2.68 (1.12)	2.57 (0.82)	2.35 (0.89)	2.37 (0.90)	3.14	0.03
**Procedural justice**	3.56 (0.96)	3.13 (82)	3.23 (0.94)	3.02 (0.89)	6.36	0.001
**Interactional justice**	3.84 (0.96)	3.70 (0.84)	3.60 (0.95)	3.67 (0.94)	2.96	0.03

^a^ Including Emergency Department, CCU and ICU

**Table 4.** The Results of Tukey Test Regarding Perception of Justice in Different Wards^a ^

Dependent Variable	(I) Ward	(J) Ward	Mean Difference (I-J)	SE	P value	95% Confidence Interval	
						Lower	Upper
**Overall organizational justice**	Surgical	Critical care^a^	0.29^a^	0.10	0.01	0.04	0.53
		Neurology, Oncology, Burn, Transplant	0.40^a^	0.12	0.005	0.09	0.71
**Distributive justice**	Surgical	Critical care	0.32^a^	0.11	0.02	0.03	0.62
**Procedural justice**	Surgical	Critical care	0.33^a^	0.11	0.01	0.05	0.61
		Neurology, Oncology, Burn, Transplant	0.54^a^	0.14	0.001	0.18	0.90
**Interactional justice**	Surgical	Neurology, Oncology, Burn, Transplant	0.38^a^	0.14	0.04	0.02	0.74

^a^ Insignificant relationships are excluded, and only the significant ones are presented

## 5. Discussion

Results of the current study showed that perception of justice in nurses with different demographic characteristics were not significantly different regarding the type of hospital. Therefore the first hypothesis was rejected. However, the mean score of perceived justice was somewhat higher in men than women. The findings were consistent with the results of Golparvar and Arizi who studied men and women’s attitude toward the world fairness (32), while Jafari and Bidarian reported no significant association between gender and the perception of justice (1). Perhaps men have lower expectations in their life and consequently feel more justice than women. The findings of this study showed that participants with nursing diploma had a perception score of OJ somewhat higher than Bachelors. Results of some of previous studies are consistent with this finding (1, 33). It seems that when people continue their studies they tend to seek better positions and be treated more respectfully, and expect more justice in the organization compared to the others with less education. In the current study, most nurses had a perception score equal or higher than the cut-off point of OJ. However the mean of justice in specialty hospitals was higher than general hospitals. Greenwald et al. showed that patients in specialty hospitals were more satisfied with nursing care received, than those in general hospitals, which is consistent with the results of the present study (34). Huang et al. also showed that fairness perceptions is affected by the type of hospital (35). Perhaps the conditions of specialty settings such as management support or inflexible work hours (36) make nurses to have higher perceptions of justice than their counterparts in general ones. Also, the mean score of procedural and interactional justice were higher than the cut-off point. It means that nurses felt justice in procedures and interactions in their organizations. In addition, of the three subscales of OJ, procedural justice had the highest score both in general and specialty settings. Robbins argued that in high perceptions of procedural justice, employees look up positively to their supervisors, even if they are dissatisfied with their salaries, job opportunities, and other personal variables (37). Some studies have revealed that emotional reaction to the organization is mostly predicted by procedural justice. However, trust in supervisor is more related to interactional justice (38). The mean of distributive justice one of the three components of OJ, in the current study, was the lowest both in general and specialty settings. Nurses felt injustice in the way the outcomes were distributed. This finding is consistent with the results of Hepnoiemi et al. and Ambrose et al. who have studied the association between justice and attitudes (6, 7). Besides, of the three subscales of OJ, all but distributive justice were significantly higher at general and specialty hospitals, It seems that the atmosphere of specialty settings compensates the difficult terms of nursing and makes nurses feel more fairness in the organizations. Therefore the second hypothesis of the study could be accepted. The findings of this study suggested that there are significant differences in perceived OJ among nurses in different wards. It seems that working in places in where nurses are faced with wounds and discomforts of patients affects the nurses’ perception of justice, even if the fairness is being considered, and justice is perceived by their counterparts. Then, it can be said that nurses working in different wards have different perceptions of OJ, and the third hypothesis could be accepted then. To sum up, the findings of this study suggested that nurses working at specialty hospitals had a more positive perception of justice than their counterparts in general settings. Nurses are the key employees at health care organizations and due to their very close relationship with patients, their perception of justice may affect the quality of care; therefore, should be considered seriously. Since nurses’ perception of injustice would lead them to become unproductive or make decision to leave their job, the direct and final effect of these consequences would be on patients. As a result not only the order of treatments or health care services would be interrupted, but also many resources would be perished. Thus, in highly demanding and stressful situations, such as healthcare services, high perception of OJ may help an employee to cope with such a situation. Our study had some limitations, first, OJ was a self-report measure in this study, although self-report data are usually used to measure job attitudes; researchers should take into account that they may not reflect the actual attitudes of the respondents. Furthermore, this research was performed within a single industry in one geographical area, so generalizability of the results may be limited. For these reasons, it is recommended to perform further investigations in different industries and sampling from different locations and occupations.
